# Reviews and Book Notices

**Published:** 1878-10

**Authors:** 


### Reviews and Book Notices.

Lectures on Clinical Medicine. By Dr. M'Call Anderson, Professor of Clinical Medicine in the University of Glasgow. London: Macmillan & Co., 1878. Chicago: Jansen, McClurg & Co.

The author, in this volume, has thrown together, in a small compass, much of what has been the result of considerable experience, reading and reflection. It consists of seventeen lectures on topics of surpassing interest to the medical practitioner.

A clear and faithful exponent of his subject, the writer presents it with perspicuity; not discolored by some pet theory, nor influenced unduly by any prevailing fashion of thought. This little work is quite *au courant* of the most advanced medical literature; for whilst the author avails himself of the learning of such men as Graves, Watson and other eminent cultivators of medical science, belonging to an age, now nearly passed away, he loses no opportunity of profiting by all that is new and progressive in science. He therefore fairly estimates such advantages as we are supposed to derive from improved instrumental appliances in guiding us to a correct diagnosis; and gives us the result of his experience as to the worth of new modes of treatment. As we read each case, his style is so simple and invested with such an air of candor, as to invite confidence even from those who are disposed to scepticism, in these times when theories are so often rashly formed, and readily pass from speculation into practice.

We propose now to give a general sketch of Dr. Anderson's work, and whilst passing in review the chief subjects of which it treats, we regret that neither time nor space will permit us to dwell on them to the extent each is so justly entitled. We must therefore limit ourselves to a very brief notice of some; and select for a fuller consideration, others, that just now engage more especially, the attention of the profession.

The first lecture is introductory. In this he shows how important clinical teaching is in making skillful and successful physicians. How, without it, the mere reading of systematic works can never reach this result; and that a chief cause of our ranks being thronged by incompetent men, is, in a great measure, due to the neglect and inefficiency of clinical instruction. In this chapter he enumerates many of the aids we derive in treatment from the simple application of cold in fever and in localized inflammation. When alluding to the cold bath, in enteric fever, he graphically describes his feelings, when for the first time, he had recourse to it as a remedy.

"I must confess," he writes, "that on the first occasion on which I used it, it was with fear and trembling. The first case in which I tried this treatment (a case of enteric fever), was a most unpromising one. The patient, a young female, had a temperature varying from 104° to 105.4°; a pulse of 134; respirations, 48. She was delirious, bordering upon insensibility, in a state of great prostration, with profuse diarrhœa, and with very pronounced congestion of the lungs. Before leaving the bath, her temperature had fallen to 101.3°, her pulse to 120, her respirations to 42. Her delirium was gone, her cheeks less flushed, and she expressed herself as feeling more comfortable. From this time onwards, all the distressing symptoms rapidly subsided, and she made a perfect recovery."

With the same object he advises the application of iced cloths. In this we ourselves, after a struggle with long-established and deeply rooted prejudice, now, from the experience of the last year or two, fully concur. He gives a case in which congestion and tenderness of the liver was thus treated by the systematic application of iced cloths to the hepatic region for half an hour three times daily, and in which the result was, as he expresses it, "magical." No blue-pill, no cupping, no leeches, nor blistering, but the simple application of cold. This treatment we have fully tried with the same favorable result.

The second lecture consists of "Cases illustrative of pain as a symptom of disease." In this he impresses on the class the circumstance that the significance of this symptom is "exceedingly varied, and that we must endeavor in all cases, if possible, to ascertain the nature of the morbid state of which it is the expression, before devising measures for its removal." But that there are some cases in which it is not possible speedily to ascertain this, must be consonant with the experience of every physician; and too strict a compliance with the rule would often be attended by unnecessary suffering to the patient. Still, we must not, from this observation, be supposed as endeavoring to invalidate a sound principle of practice. For in all cases we should investigate and try to ascertain the true meaning of pain, no matter from what part it may proceed, just as we would interrogate the abnormal functions of any organ, with the view to ascertain the relation that the derangements of the former bear to the latter.

The third lecture is devoted to cases illustrative of gastric and cerebral vomiting. In this he contrasts their distinctive features and presents two cases as types of their respective kinds. The eighth and ninth lectures are of paramount interest and importance. They are models of excellence in clinical teaching. The subject of both is the "Treatment of aneurism of the aorta." Cases of such are graphically related, followed by comments clear and condensed. In the treatment, the author deviates, not unjustifiably, however, from what has been the routine, but we must add, hitherto unavailing practice in a large majority of instances.

Our readers may remember that with Morgagni commenced the era of clinical teaching of aneurism of the aorta. For though the credit of distinguishing the disease in the living subject, and proving the correctness of the diagnosis by subsequent autopsy, belongs to Vesalius, who wrote in 1557; yet more than a century elapsed before Morgagni considered the subject in the more copious recital of facts; in his clearer views respecting the nature and mode of formation of aneurismal tumors; in his exposition of the morbid changes of the vessel inducing them; in his demonstration of the many and varying manifestations that arise from their compression of surrounding parts; and in his accuracy in the enumeration of their several terminations. Since then, Scarpa, Corvisart, Lænnec, Bouillaud, and Hodgson have contributed, each his share, to throw light on a subject that had been so little understood that Riolan, in 1658, declared that aneurism could not affect the aorta, owing to the thickness of its walls, and Elsner, in 1690, when writing of a case of aneurism of the aorta, refers to it under the head "De paradoxico aneurismate aorto." Still, nearly half a century ago, previously to the writings of Stokes, Greene and others of the Dublin school, clear and distinct views derived from anatomy and physiology were not generally held on this disease; so much so, that we can remember when a correct diagnosis of aneurism of the aorta was considered among the especial difficulties of practice. At present, what a marked contrast! What aids we acquire in closely observing pathological phenomena, as in the progress of the affection, the tumor extends, and comes into close relation with surrounding parts! Here our knowledge of anatomy and physiology enables us to grasp every indication presented, as each organ in turn invaded by the tumor supplies us with varying manifestations progressively developed.

Aneurism of the aorta arises most frequently at the origin of that vessel, and here it is in relation with the heart itself. It may compress the auricles, the pulmonary artery and the superior cava; increasing, it presses on the trachea, the bronchi, the œsophagus, the pneumogastric, and the recurrent nerves, particularly the left recurrent. Coming forward it perforates anteriorly the thorax and appears externally. It may perforate a lung, or open into the cavity of the pleura. As each function is interfered with, symptoms arise, whether as regards the viscera, the circulation or the nerves, the import and interpretation of which must be determined by our knowledge of the structure and functions of the organism. It must not be supposed that the disease terminates, after perforating the thorax, in hæmorrhage, in the majority of cases. Asphyxia is the most common cause of death. When aneurism of the arch of the aorta presses on the bronchi, trachea, on one of the lungs, or on the pulmonary artery, this most likely will be the result. After asphyxia, hæmorrhage is next in the order of frequency; then inanition by compression of the œsaphagus; gangrene of the lung; paraplegia and sloughing of the sacrum from pressure on the spinal marrow or softening of the brain by obliteration of the carotids, or finally by mere exhaustion. However, without further preliminary remarks, we will let the author speak for himself:

"On the 11th October, 1873, there was admitted into the Royal Infirmary, a man who was about 34 years of age. For the last four years he has been a French polisher, but previously to that time he was a joiner, and was frequently required to lift heavy weights. His diet has always been good, and his habits temperate. He has uniformly enjoyed good health, and has had neither rheumatism nor syphilis. About three years ago he began to complain of palpitation, especially on exertion, and 21 months after this, while attending his wife, who was laid up with fever, he experienced a sharp pain in the left breast, which extended into the left shoulder and down the arm. On account of these symptoms, he entered the infirmary on January 11th, 1873, and was said then to be laboring under dilated hypertrophy of the heart. Under the influence of rest, tincture of veratrum viride in 5-drop doses, and syrup of the iodide of iron, he improved considerably, and was dismissed on March 29. For four months after this he continued in tolerable health, although he suffered at intervals from the palpitation and pain; but about three months ago he observed that he was becoming hoarse, and since then the hoarseness has gradually increased, although it never amounted to aphonia. About this time, too, he commenced to complain of attacks of dyspnœa, coming on for the most part when speaking or walking rapidly, and giving rise to a feeling of suffocation referred to the region of the larynx. For some months also he has had a short, dry, somewhat hollow cough, unaccompanied by expectoration. About five weeks prior to admission, a pulsating tumor was detected by a medical man in the jugular fossa, but for a couple of weeks before this he felt a beating above the breast-bone. When the tumor made its appearance, the palpitation and pains in the chest subsided. When first discovered it formed a well marked prominence at the root of the neck, was about the size of a hen's egg, and was tender to the touch. After the appearance of the tumor, some difficulty of swallowing was experienced, and he had the feeling 'as if the food was going the wrong way.' This symptom has latterly in great measure disappeared. He does not sleep well at night, which seems to be due to the pulsation in the tumor. He perspires freely, has been losing flesh rapidly, and frequently changes color. Tongue moist and slighted coated, appetite variable; bowels uniformly costive; pulse 66, natural; temperature, 98.4°." "On uncovering this man's chest, and placing him upon his back, a distinct swelling was observed at the top of the sternum, and inclined a little to the left side—a swelling which was not very prominent, but which occupied an area about equal to that of a hen's egg. It was to the eye a distinctly pulsating swelling; and on applying the hand, it was found to be very soft, and not only the seat of pulsation, but also, what is a very characteristic symptom, of expansion, and each time the ventricle contracted a distinct vibration was experienced—'purring tremor,' as it is called. On percussion, there was well marked dullness, not only over the tumor, but also over the upper part of the manubrium sterni, and extending a little into each subclavicular region. On applying the stethoscope a loud, rasping, systolic murmur was heard over the tumor. It was also heard over the whole of the chest in the vessels of the neck and arms, although more markedly in those of the right than in those of the left side, and in the thoracic and abdominal aorta, but it was inaudible in the femoral vessels. The history of this case and these physical signs pointed very conclusively to the existence of an aneurism of the arch of the aorta.

"But let me point out to you before passing further that in cases of intra-thoracic tumor you may have a somewhat similar history, and you may have likewise most of these physical signs, and, therefore, mistakes are apt to be made. You may even have the physical signs of pulsation and of murmur; of pulsation, because the tumor may be lying on the aorta, or one of the large vessels from which it may be communicated; and of murmur, because it may be pressing on one of these vessels and obstructing the flow of blood through it. There were, however, two physical signs in this case which are not observed in cases of tumor. One of these is 'purring tremor,' and the other the feeling of expansion in addition to pulsation. Then there were other features in the case which confirmed our diagnosis, if such confirmation was needed. On examining the superficial vessels of the arm for example, we observed that they pulsated very visibly; that they were tortuous, and felt like firm, hard, almost tendinous cords. In fact, we had here signs indicating a degeneration of the coats of the superficial vessels; and we know that when the superficial vessels are degenerated, the coats of the aorta are generally degenerated, too, and that a degeneration of the coats of the aorta is the usual predisposing cause of aneurism. Then on examining the heart we found that the apex-beat was displaced. It was situated three and a half inches below, and an inch and a half to the left of a vertical line drawn through the nipple. The impulse of the heart was heaving; it was observed over a preternaturally extensive area, and there was increased dullness on percussion in a downward direction, and to the left. That is to say, there were the usual symptoms of dilated hypertrophy of the left ventricle of the heart—a condition which is very usually, though not invariably, met with in connection with aneurism of the aorta, owing to the obstruction which it offers to the onward passage of the blood. In cases of aneurism, too, and for a similar reason, we often find inequalities in the pulses on the two sides of the body. There was not much difference in the pulse at the wrists in this case, but in the right carotid the pulsation was very much stronger than in the left. Again, in non-aneurismal tumors, and particularly in cancerous tumors, which are those most frequently observed within the chest, there is very often a marked distention of the superficial veins. In aneurism this is not generally a marked feature, because the aneurismal tumor is soft and yielding, and does not compress the parts to the same extent as a solid, and therefore unyielding, tumor. In our patient there was no marked distention of the veins. And, lastly, the sex of the patient was just what we might expect in a case of aneurism, which is much more frequent in males than in females. There are, however, certain symptoms frequently observed in cases of aneurism of the aorta, which were conspicuous (sic) for their absence in this one. Let me mention a few of these. Sometimes the thoracic duct is compressed, and then, owing to the system being deprived of its nutrient supplies, there is rapid emaciation. Our patient was not much emaciated. Again, in a case in Dr. Scott Orr's wards, in which I lately performed galvano-puncture, there was dilatation of the left pupil from pressure upon the sympathetic, which was absent in our patient. In a good many cases there is pressure upon the recurrent nerve; but in this one, although the patient was hoarse, there were none of the usual symptoms of pressure upon it; there was no paralysis of the vocal cords, and there were none of those attacks of suffocative dyspnœa which constitute such a distressing feature of many cases of aneurism of the aorta. And, finally, there was no evidence of distinct pressure upon the bronchi, because there was no marked shortness of breath, and the air entered apparently with equal freedom into both lungs.

This patient was admitted on the 11th of October, and we thought it right, for a short time at all events, to watch him without carrying out any very energetic treatment. We kept him in a state of perfect repose in bed; we regulated his bowels with castor oil in order to prevent any straining at stool, and, with the view of calming down the nervous and circulatory systems, we gave him five and twenty grains of chloral at bed time. It soon became evident, however, that the tumor was progressing rapidly towards the surface, and the only hope for him, therefore, in my opinion, lay in giving him the chance of the operation of galvano-puncture. This was performed seven times in all—namely, on October 22d and 26th, on November 2d, 7th and 23d, and on December 1st and 14th. In each operation a Stohrer's battery with large cells was used; and unless I state the contrary, you may take it for granted that only one needle was used, and connected with the positive pole. The first operation then was on the 22d October, on which occasion eight cells were used, and the operation lasted half an hour. On October 26th the tumor was not in the least diminished, but, on the contrary, was becoming more prominent towards the left side, and the patient complained of a feeling of soreness across the root of the neck. On this day, therefore, the second operation was performed. Six cells were used for ten minutes, and then eight for fifty minutes—one hour in all; and during the whole of this time the patient complained of burning heat in the aneurism. On October 28th the tumor was somewhat firmer, and a distinct hard line was observed along the track of the needle. The skin covering it was the seat of a slight inflammatory blush, and the patient complained of a feeling of stiffness across the root of the neck, and occasionally of "stounding" pains in the tumor itself; temperature 99.3°. On account of these symptoms of reaction we only allowed him light food—milk diet and soup. We gave him a draught of castor oil, and applied ice-cold cloths over the tumor for half an hour at a time. On October 30th the pain in the tumor had disappeared, and the temperature had fallen to 98.4°, but the feeling of stiffness and soreness was even more marked, and there was some complaint of pressure upon the windpipe, of shortness of breath, and of weakness. On November 2d the third operation was performed, the needle being introduced into upper part of the tumor. Eight cells were employed on this occasion, and the operation continued thirty minutes, during the whole of which time the patient complained of intense burning pain. This, however, was soon relieved, as on the former occasion, by the application of iced cloths. On the 3d November, as he complained a good deal of palpitation and of pulsation in the swelling, the tincture of veratrum viride was prescribed in doses of three drops, gradually increased to ten, three times a day. On the 6th November the tumor felt firmer. There was no pain, and the patient felt comfortable. On the following day the fourth operation was performed. On this occasion two needles were made use of—one connected with the positive, and one with the negative pole. They were introduced parallel to one another, and an inch and a quarter apart. The operation was continued for thirty minutes, four cells being used for the first twenty, and six for the remaining ten minutes. During the whole time the patient complained of intense burning pain. Now mark what happened on withdrawing the needles. On withdrawing the needle connected with the positive pole, not a drop of blood escaped; on extracting the needle connected with the negative pole, however, a jet of blood followed, which at first was dark, but soon became florid and spouted in jerks as from an artery of moderate size. Nearly an ounce escaped in all, and the hæmorrhage was arrested by the pressure of a graduated compress of lint. I shall refer to this again. On the 23d November it was reported that the patient had had no pain since the last operation, and that the heart's action under the influence of the veratrum viride was calm. On examining the tumor on that day it was found to be less firm and more prominent, and in the center there was distinct pointing, and the skin in this situation had a deep brownish-red tint. The fifth operation was peformed on this day. One needle was used, which was introduced into the central part of the tumor, the point of the needle being immediately opposite the part which was pointing. Six cells were used, and the operation was continued thirty minutes. Burning heat was complained of, but this was relieved as before by the application of iced cloths. After the operation the pulse was 85, and the heart's action excited, but this was relieved by the administration of ten minutes of the tincture of veratrum viride. On the 1st of December the tumor was observed to be much more prominent and pointing most distinctly, and the discoloration of the skin had much extended. Galvano-puncture was performed for the sixth time; six cells being used for fifty minutes. On the 10th of December my assistant, Dr. Strother, being summoned to the patient, found that there was a slight oozing of bloody serum from the most prominent part of the tumor. It was arrested by the application of collodion. On the 14th of December the seventh and last operation was performed. Three needles, which were not insulated, were used on this occasion, all in connection with the positive pole, the needles being inserted so that the points were opposite the most prominent part of the swelling. Six cells were used, and the operation was continued for an hour. During the operation there was a slight oozing of blood, owing to the displacement of the collodion. On the 15th of December, at half-past four o'clock in the morning, a considerable stream of blood was observed to be flowing down the chest, having separated the collodion covering. The whole tumor was enveloped in narrow strips of lint, soaked in collodion. Over these, strips of gutta-percha tissue were placed and sealed up with chloroform, which for the time arrested the bleeding. Next morning, at half-past seven, the patient became very weak and vomited. Dr. Strother placed his hand upon the tumor in order to give it support, and while the patient was retching something distinctly gave way, and he exclaimed, "There it is!" This was followed by a gush of blood on all sides, which quickly saturated the sheet and pillows, and for a time he was pulseless. The hæmorrhage was again arrested as before, and the vomiting ceased. After the administration of iced champagne the patient rallied a little, but from time to time the bleeding recurred, and he sank, exhausted and insensible, on the 18th of December, at 10:30 a. m.

On post mortem examination, the surface of the body presented nothing remarkable, with the exception of a dark prominent mass, which protruded from the skin immediately above the supra-sternal notch, and inclined a little to the left side. It had an oval shape, and measured about three inches from above downwards, and two inches across. Its surface was very irregular, and it was evidently composed of coagulated blood. It was found to communicate, by an aperture in the skin, measuring two inches from above downwards, and one inch across, and about the level of the thyroid gland, with an aneurism of the aorta. The heart was very much enlarged, the left ventricle in particular being hypertrophied and dilated. Owing to the way in which it was thought right to remove the parts, its exact weight could not be ascertained, but it probably weighed from twenty to twenty-two ounces. Numerous calcareous plates were found in the aortic arch, which was somewhat wider than natural. A very large, sacculated aneurism sprang from the upper surface of the transverse portion of the arch, with which it communicated by an apperture large enough to admit two fingers, and situated just before the giving off of the large vessels, none of which were directly involved in it. The aneurism was somewhat oval in shape, its long axis, directed from above downwards, measuring between five and six inches. It lay in front, and a little to the left of the larynx and trachea, which it forced slightly backwards and to the right, but it did not seem seriously to interfere with these parts. Although the large vessels were not at their origins involved in the aneurism, the left carotid and subclavian were found to be firmly adherent to, and in part imbedded in, the walls of the sac, while the innominate was only very slightly attached to it. The sac was almost completely filled with old and recent clots, and at one place, almost in the middle of the sac, and in a line with one of the needle punctures, there was a very distinct stratified coagulum which was much paler and firmer than the rest. The other organs of the body were healthy."

This lecture is of great interest, both in a scientific and practical point of view, and merits the attentive consideration of the profession. We cannot avoid, however, impugning what, in two instances, we believe to be inaccuracies. Dr. Anderson tells us that "in a case in Dr. Scott Orr's wards, in which I lately performed galvano-puncture, there was *dilatation* of the pupil from pressure upon the sympathetic." Now, we can understand how dilatation of the pupil may arise from *irritation* of the sympathetic, owing to the proximity of an aneurismal tumor: but simple compression of the sympathetic would have quite the contrary effect. It would cause contraction of the pupil. As far back as the close of 1851, M. Claude Bernard made public the results of his experiments on the effects of section of the cervical sympathetic; one of the most marked of these being the permanent contraction of the pupil. Even as far back as 1727, Pourfour du Petit fully established the fact that the radiating fibers of the iris are under the influence of the sympathetic system, or, in other words, that the sympathetic acts as a simple conductor in transmitting an influence seated in that portion of the medulla spinalis, extending from the first to the sixth dorsal vertebra, which portion has been termed by Budge, the *cilio-spinal region.* But more recently Bernard determined accurately the region from which the sympathetic derives its influence upon the pupil, and is of opinion that it resides in the portion of the medulla included within the two first dorsal pairs; and that it is transmitted to the ganglionic system more especially by the anterior branch of the second pair. Banks (*Dublin Hospital Gazette*, 1856) relates a case of aneurism of the aorta, in which there was a permanent contraction of the left pupil from compression. Hane, in 1838, Gairdner, (*Monthly Journal of Medical Science*, Feb., 1853), and Williamson in 1857, all proved the same effect of compression of the sympathetic in aneurism of the aorta. Somewhat further our author observes, "In a good many cases there is pressure upon the recurrent nerve; but in this one, although the patient was hoarse, there were none of the usual symptoms of pressure upon it; there was no paralysis of the vocal cords." Here we are disposed to draw the very opposite inference from the circumstance of hoarseness as stated. We would consider it as an indication of partial pressure, or pressure limited to one or other recurrent. Where there is compression of both recurrents, we expect to find complete aphonia.

Traube (*Deutsche Klinik*, 1860, No. 41, and 1861 No. 27) has published two cases of this disease, in which the larynx was examined by means of the laryngoscope. In one the voice was not entirely lost, and paralysis was limited to the left recurrent. When the patient would pronounce the letter e, the left vocal cord remained immovable. In the other case there was complete aphonia; the glottis was largely dilated; the left vocal cord was altogether immovable; the right slowly approached the median line. Mere feebleness of voice in aortic aneurism generally depends on pressure on the trachea; but where there is hoarseness, or complete aphonia, there is compression of one or other recurrent, or of both at the same time.

We may seem to be hypercritical in thus adverting upon what will seem as blemishes, trifling though they be, in comparison with what otherwise is, in a high degree, deserving of commendation, but we trust we are governed solely by a sense of what is due to just criticism—in demanding the pointing out of defects equally with the praise of excellence.

The remaining lectures are valuable, and will amply repay perusal. We regret that space compels us to draw our strictures to a close, and we take leave of the author on this occasion, in recommending his work as an excellent guide either for student or practitioner. W. G. D.

LESSONS IN LARYNGOSCOPY, INCLUDING RHINOSCOPY AND THE DIAGNOSIS AND TREATMENT OF DISEASES OF THE THROAT. By Prosser James, M. D., M. R. C. P., etc. Second edition, with colored plates. Baillière, Tindall and Cox, London, 1878.

This is a small book of 176 pages, the first edition of which was issued in 1873. The author has not thought it necessary to revise the text for the second edition, but has merely added several colored plates, which represent with considerable accuracy various conditions of the larynx, and will be of benefit to beginners. The work is freely illustrated with wood-cuts, which, owing to careful descriptions, answer their purpose well, and the author's style is so simple and lucid that his instructions may readily be understood by the student.

The author, after a brief history of laryngoscopy, describes the various instruments and apparatus required, and the proper methods of using them. He then describes the normal laryngeal image, and in the fourth chapter points out the difficulties which the laryngoscopist has to encounter, and gives plain and simple directions for overcoming them.

Then follows a more minute description of the larynx and the various modes of demonstration, all of which is rendered intelligible even to the tyro. In the eighth chapter Rhinoscopy is considered, and in the tenth the Practical Use of Laryngoscopy in diagnosis and treatment of the various diseases of the vocal organ.

This little work is the most valuable guide we have seen for the student of laryngoscopy, and we can heartily recommend it. Its excellencies are many, and its defects unimportant, though we confess that the wood-cuts and press-work are the poorest we have ever seen in a medical work. It is very unfortunate that a work so well written should have appeared in such a shabby dress.

Ninth Annual Report of the State Roard of Health of Massachusetts. January, 1878.

Besides a great many statistics and reports of local interest, this volume contains a few papers which it is a pity to bury in a book of such limited circulation.

Dr. J. F. A. Adams, of Pittsfield, gives the description and plans of "Cottage Hospitals," as they have been established in many country places in England ever since 1859.

Professor Wm. Ripley Nichols contributes a lengthy article on the "Filtration of Potable Water." In regard to artificial filtration on a large scale, the following conclusions are arrived at:

1. No other material has yet been brought into practical use for artificial filtration on a large scale, except sand.

2. With our present knowledge, we have no evidence that sand filtration can be regarded as an efficient means of purification of *polluted* water; although it may, if properly carried out, lessen the liability of ill effects.

3. All visible suspended particles, and an appreciable proportion of organic matter actually in solution, may be removed by properly conducted filtration through sand.

4. For the present, at any rate, it will be best to regard artificial filtration mainly as a means for the removal of *suspended* matter.

The so-called method of natural filtration consists in drawing the supply from what is known as the ground-water, and is practicable only in localities where the ground-water is of good quality, and free from possibility of pollution.

The most interesting paper in the volume is Dr. B. J. Jeffries' essay on the "Dangers arising from Color-blindness," with especial reference to railroad employés and sailors. Just about one hundred years ago the first cases of color-blindness were made public by Joseph Huddart. In 1794 the English chemist Dalton published an account of his own case, he being red-blind; and ever since red-blindness was called Daltonism. Dr. George Wilson, of Edinburgh, in 1855, in his book on color-blindness, has already referred to the dangers from this defect of vision. He says: "The professions for which color-blindness most seriously disqualifies, are those of the sailor and railroad servant who have daily to peril human life and property on the indication which a colored flag or lamp seems to give. * * * The appalling yearly list of lost vessels awakens the suspicion that more than one of these fatal disasters may have resulted from the mistaken color of a light-house, beacon, or harbor-lamp.

"On railways the danger attending mistakes of signals is much greater than at sea. The most marked peculiarities of the color-blind are shown in mistaking (1) bright red for green, (2) dark red for brown, (3) red for black, and (4) dark or light shades of all colors for each other. The caution signal *green* is thus liable to be mistaken for the danger-signal *red*, and the latter (when it appears black) not to be seen at all."

But Dr. Wilson's voice of warning was not heeded, and his efforts did not bear the fruit they deserved. Twenty-two years later, in 1875, a railroad accident occurred in Sweden, which greatly excited public attention and the evidence of which lead Prof. Holmgren to suppose that color-blindness was one of the principal causes of the disaster. He gave this matter his whole attention, and by untiring efforts succeeded at last, that in 1876 throughout Sweden it was ordered that all the railroad employes should be tested for color-blindness by Holmgren's method. This method of examination combines simplicity and rapidity with absolute certainty; it consists in the use of worsteds of various colors whereby the person examined does not name the colors, but simply selects them by comparison with the test. By it, a number of eyes can be tested for color-blindness in a relatively short time (about one minute for one person) whilst it does not call for any special intelligence on the part of the person examined.

The magnitude of the danger from color-blindness depends, of course, on the frequency of the defect. In general it may be said that the more accurate examinations of recent date show it to be more frequent than one is generally inclined to think. Dr. Wilson tested altogether 1,154 persons, and found 65, or 5.6 per cent. color blind. Prof. Holmgren found 13 color-blind among 266 railroad employes, or 4.8 per cent. Prof. Dove, of Berlin, reports 40 color-blind men among 860, or 4.6 per cent. Dr. Favre, in France, up to 1855, examined 5,000 candidates for railroad work, and rejected more than 50 for being red blind, and among 224 conductors examined by him, 14 were found decidedly color-blind. Prof. Donders examined 2,300 employes on the Holland roads and found 152 of these color-blind. Dr. Jeffries has recently tested 611 instructors and students of Harvard University, and detected 30 color-blind.

The best proof of the recognition of the great danger from color-blindness is the fact that measures have been recently taken to test men for color-blindness on most of the railroads in Sweden, Denmark, England, France, Germany, Holland, Prussia and Italy. Nothing has yet been done in this country; this whole question was first publicly discussed in 1877, before the Boston Society of Natural History, through the efforts of Dr. Jeffries. Thanks to his persistent labors, we are informed, several railroad companies have already ordered an examination of all their employes for color-blindness. And let us hope that the time will not be far when every railroad company will be compelled by State laws to have every applicant for service examined for color-blindness by an expert and to refuse him if disqualified.

F. C. H.

PRESCRIPTION WRITING. Designed for the use of medical students who have never studied Latin. By Frederic Henry Gerrish, M. D., Professor of Materia Medica and Therapeutics in the Medical School of Maine, etc. Second edition. Portland Me.: Loring, Short & Harman. 1878; pp. 51.

The object of this little book is to give to those who have never studied Latin, what little knowledge of this language is essential to correct prescription writing. It contains such general rules for writing prescriptions, as should be observed, whatever the language may be in which the prescription is written; it shows the variation in Latin words in the different declensions necessary to indicate the possessive and accusative cases; and to illustrate the rules, a few examples are given of prescriptions written in English and translated into Latin, the translation being explained step by step in detail. In the second part of the book are collected all the Latin words used in the United States Pharmacopœia. The nouns and adjectives are classified in five lists according to their declensions; the sixth list contains the indeclinable nouns; list VII gives the numerals and ordinals; list VIII the verbs, and list IX the adverbs, conjunctions and prepositions.

The whole subject of writing prescriptions in Latin is reduced to its smallest possible compass. And as long as the fashion of Latin prescription writing is continued in text-books, journals and practice, so long will this little book prove useful (we dare say indispensable) to a great many American physicians who care to conceal their lack of classical education, and to spare the feelings of medical editors, so often offended by manuscripts bristling with imperfectly written formulæ. F. C. H.

A MANUAL OF NURSING. Prepared for the Training School for Nurses, attached to Bellevue Hospital. New York: Putnam's Sons. Chicago: Jansen, McClurg & Co. Pp. 143; price $1.00.

This little work has been prepared in the hope that it will be useful to those who are earnestly studying to become good and efficient nurses. It contains terse and clear instructions on hospital management, observation of the sick, administration of medicine, applications, dressing of wounds, operations, special medical cases, emergencies, monthly nursing, nursing of sick children, hygiene of children, with rules for cooking for the sick, and table of weights and measures.

Many ideas are borrowed from Miss Nightingale's "Notes on Nursing;" also from Miss Lee's "Hospital Sisters," proper credit being given to both of these authors.

The work cannot fail not only to meet the object intended for the class specified, but has in it much to recommend it to the perusal of a still larger class—to the mothers and attendants in homes where poverty denies the admittance of the professional nurse.
S. F. B.

VISIONS; A STUDY OF FALSE SIGHT (Pseudopia.) By Edward H. Clarke, M. D. With an introduction and memorial sketch by Oliver Wendell Holmes, M. D. Boston: Houghton, Osgood & Co., 1878; 12 mo. pp. 315.

This is a remarkable book. It is said to be an unfinished essay but most readers would fail to discover the fact in reading the work.

Written while the author was waiting to die of cancer and for occupation during this weary time, notwithstanding the perturbations of mind that must have attended the labor, it appears in the author's most fascinating style and to the end of the book it contains no internal evidence of failing faculties. What a man — what a mind, to have wrought such a work under such circumstances!

The essay is an attempt to find a physiological explanation of pseudopia—false sight—of visions.

Seven instances of the seeing of visions are detailed. In each case the subject was a cultivated and trustworthy person and one also who, while he beheld the unreal object, knew the sight was false, knew it must be only an appearance; in some cases knew or believed the cause to be in his own disturbed brain or body. Having related these examples, the author proceeds to discuss the physiology of sight and dwells at length on the rôle played in this function by the different parts of the cerebral machinery.

The discussion is carried into the field of pathology of this function and the conclusions arrived at—or some of them—may be fairly represented by a few quotations from the writer's own words:

"Seeing is a matter of the brain and not of the eye; the eye only transmits impressions."

"Human sight is not accomplished till sensory impressions are transformed into ideas and this is done in the hemispheres."

"When this is done—when the organic basis of visual ideas is formed there, seeing takes place, whether there is any corresponding external object or not."

"The brain cells, acting under subjective stimuli, may arrange themselves in such a way as to represent a vision, that is sight, when no external object, corresponding to it, exists."

"A vision is produced whenever the cell-groups, indicating that vision—its hieroglyphic or cypher—are formed in the brain, whether they are formed normally, by the stimulus of light waves from an external object, or abnormally, by a stimulus initiated intra-cranially."

Whether all of the ideas of Dr. Clarke about the details of cerebral physiology are correct we do not need to examine. His notions of the function of the tubercula quadrigemina, of the angular gyri, of the hemispheres, in the physiology of sight, may in the future find themselves far from the accepted doctrines—to-day they would doubtless be questioned by some. But it must be true that normal sight is attended with some change of a peculiar and characteristic kind in the brain. Whether this be called by the name of cell-grouping or by some other, matters little. This admitted, the conclusions of Dr. C., in explanation of visions seem beyond controversy. They are not only correct to-day, but they are of that character that compels the belief that they will be true in the light of the knowledge of the future.

In thus explaining, in a philosophical manner, the appearance of visions, in an essay almost as acceptable and readable to those outside as in the medical profession, our author has done a substantial service to mankind.

The measure of this service is fully understood only when we consider that the explanation of visions is directly or indirectly the explanation of much of the manifestations of modern spiritualism, of trance, somnambulism, and the like. The conclusions of the author prick many of the bubbles of this kind, blown so industriously, and for so long, by a great multitude of very well-meaning spiritualists and believers in modern wonders and miracles, some of whom have gone crazy, or worried their lives away on account of the sights they have supposed they or others have seen. N. B.

St. Bartholomew's Hospital Reports, Vol. XIII, 1877, Edited by W. S. Church, M. D., and Alfred Willett, F. R. C. S.

This valuable work presents, as usual, an admirable collection of papers by members of the hospital staff. The volume opens with a paper by Dr. Andrews, "On Presystolic Murmurs at the Heart's Apex," giving at some length the characters of the sound and nature of the lesions which accompany it; subjects upon which the author has some views of his own. The second paper is by Dr. Legg, upon "The Essential Features of Jaundice," in which he describes quite fully the invariable appearances accompanying the disease, the color, the condition of the various secretions, changes in the blood, the fæces, and the urine with its various and varying constituents and tests for the same; the pathology of the disease is hardly touched upon, still less its ætiology. References to its literature are copious, and some rare cases are mentioned. The paper is a valuable one.

Mr. Leach gives some "Brief Notes on the Outbreak of Scurvy in the late Arctic Expedition," being a summary for the Blue Book containing the testimony elicited during the admiralty examination, into the causes thereof and circumstances attending it. The crews of the "Alert" and the "Discovery" started on their long expedition exceptionally well prepared; yet sixty of them were seized with scurvy and four died. Most of those attacked were addicted to intemperance, while at the same time insufficient supply of lime-juice and living in close quarters below deck had much to do with the spread of the disease.

Mr. Kesteven contributes two papers; one (illustrated) entitled "Further Histological Researches on the Pathology of the Nervous System," the other on the "Structure and Functions of the

Olivary-Bodies." The first is for the most part a lucid description of certain changes—atrophic and degenerative—in the nervous elements and blood vessels of the nervous system. He holds, with Key and Retzius, that the peri-vascular spaces are not normal; that dilatation of a vessel will induce absorption or sclerosis of its surrounding nervous substance, and that after death the vessels, being no longer distended, contract and thus seem to be lying in these spaces. He also doubts whether the vascular endothelial proliferation described by Heubner as well as the 'miliary degeneration,' can be regarded as pathognomonic of syphilis. His description of the olivary bodies is clear and concise; he regards them as associating the functions of the fifth, eighth and ninth nerves, with whose nuclear origins they are so closely connected.

Dr. Gee furnishes three papers. One "On Chronic Pneumonia Attending Disease of the Tracheal and Bronchial Glands," with four cases; one on "Spastic Paraplegia," with four cases; and "A Contribution to the History of Polydipsia," giving a brief but interesting account of two cases of congenital and and inherited diabetes insipidus, the disease having run through four generations.

Mr. Champneys describes a case of "Extroversion of the Bladder, in a Female Child," which he makes a text for an extended account of the malady, its anatomy and ætiology, with a bibliography of the subject. The same author gives also a "Case of interstitial Fibromyoma of the Uterus," which complicated pregnancy and labor. This paper is rendered more interesting by the same resort to the literature of the subject that characterized his first.

Dr. De Havilland Hall contributes an important paper "On Fribinous Exudation in the Bronchial Tubes." He reports a case of plastic or fibrinous bronchitis, and writes at some length summarizing our knowledge, or rather ignorance concerning its pathology and treatment.

Dr. Legg reports some "Cases Bearing on Diseases of the Liver," including a case of supposed "movable liver," two cases of cirrhosis in children, and one case of primary, melanotic cancer. He appends a bibliography of his subjects, which adds largely to the value of the report.

Papers are contributed by Dr. Godson "In a Case of Paroxysmal Hæmaturia;" by Mr. Vernon on "Intolerance of Light;" by Mr. Butlin on "Fatty Tumors in Infancy and Childhood." Mr. Hussey reports a case of "Aneurism in the Gluteal Region," which was operated upon and which finally died of exhaustion and complications.

Mr. Albert Doran has an illustrated article on "Complete Intra-peritoneal Ligature of the Pedicle in Ovariotomy." He favors cutting off such ligatures close to the knot, and proves from pathological specimens that such practice is unattended with danger.

Mr. West furnishes a report of a number of medical cases, including one on "Hypertrophic Cirrhosis," which is accompanied by a lithograph of the microscopic appearances. Surgical reports are furnished by Mr. Holden on a case of "Aneurism of the Right Subclavian," for which amputation at the shoulder was performed without avail; by Mr. Eve, of a case of "Fracture through the Odontoid Process," with a collection of similar cases (eleven) gleaned from the literature of the subject; and by Mr. Baker, on "The Formation of Synovial Cysts in the Leg in Connection with Disease in the Knee-joint," which title explains itself. Dr. Duckworth in a paper on "The Ætiology of Mitral Stenosis," combats the idea that rheumatism is necessarily an essential factor, not more than 60 per cent. of his cases having such an origin. Cases are reported by Mr. Kidd, Mr. Abercrombie and Mr. Darbishire, which we have not space to notice. One of the most valuable of all the papers is "On the Albuminous Substances which occur in the Urine in Albuminuria," by Dr. Brunton and Mr. Power, which is a positive contribution to our knowledge of this subject. We regret that we cannot even sum up the authors' general results.

To the above are appended the proceedings of the Abernethian Society, including a learned address by Dr. Gee, and an interesting paper on "Delirium" by Dr. Verco.

The volume closes with elaborate statistical tables of patients under treatment, which are models in their way. The illustrations are all good, and the work fully up to the standard.

R. P.

THE IDENTIFICATION OF THE HUMAN SKELETON; A Medico-legal Study. To which was awarded the prize of the Massachusetts Medical Society for 1878. By Thomas Dwight, M. D., of Boston, late professor of anatomy at the medical school of Maine. Boston: David Clapp & Son, printers, 1878. Octavo, pp. 54.

This is an ably written essay. The subject of which it treats is presented in a clear, concise way and with a judicial fairness and care that must give to it an important place as a guide in any legal investigation wherein the identification of the human skeleton is an important question. The work embodies a fair compilation from authors on the subject—whose conclusions are discussed, but it acquires its chief value from the records of the numerous careful measurements of skeletons which the author has himself made. It is illustrated by a number of graphic drawings, some of which represent an actual scale in centimeters. We are glad to see this, for if we are to be compelled to learn the metric system, as now seems to be the case, the more we are familiarized with the new measures the better.

N. B.

LANDMARKS, MEDICAL AND SURGICAL. By Luther Holden, F. R. C. S., etc. From the second English edition. Philadelphia: Henry C. Lea. 1878; pp. 128.

This little book, originally dedicated to the students of Saint Bartholomew's Hospital, will prove to be a very useful anatomical guide-book to every student and practitioner inasmuch as a thorough knowledge of the anatomical relation of the surface-marks on the human body to deeper-seated structures, is of the greatest practical importance.

